# Paired analysis of tumor mutation burden for lung adenocarcinoma and associated idiopathic pulmonary fibrosis

**DOI:** 10.1038/s41598-021-92098-y

**Published:** 2021-06-17

**Authors:** Yasuto Yoneshima, Eiji Iwama, Shingo Matsumoto, Taichi Matsubara, Testuzo Tagawa, Keiichi Ota, Kentaro Tanaka, Mitsuhiro Takenoyama, Tatsuro Okamoto, Koichi Goto, Masaki Mori, Isamu Okamoto

**Affiliations:** 1 Research Institute for Diseases of the Chest, Graduate School of Medical Sciences, Kyushu University, 3-1-1 Maidashi, Higashi-ku, Fukuoka, 812-8582 Japan; 2 Department of Thoracic Oncology, National Cancer Center Hospital East, Kashiwa, Japan; 3 Department of Thoracic Oncology, National Hospital Organization Kyushu Cancer Center, Fukuoka, Japan; 4 Department of Surgery and Science, Graduate School of Medical Sciences, Kyushu University, Fukuoka, Japan

**Keywords:** Lung cancer, Cancer genomics, Non-small-cell lung cancer

## Abstract

Genetic alterations underlying the development of lung cancer in individuals with idiopathic pulmonary fibrosis (IPF) have remained unclear. To explore whether genetic alterations in IPF tissue contribute to the development of IPF-associated lung cancer, we here evaluated tumor mutation burden (TMB) and somatic variants in 14 paired IPF and tumor samples from patients with IPF-associated lung adenocarcinoma. We also determined TMB for 22 samples of lung adenocarcinoma from patients without IPF. TMB for IPF-associated lung adenocarcinoma was significantly higher than that for matched IPF tissue (median of 2.94 vs. 1.26 mutations/Mb, *P* = 0.002). Three and 102 somatic variants were detected in IPF and matched lung adenocarcinoma samples, respectively, with only one pair of specimens sharing one somatic variant. TMB for IPF-associated lung adenocarcinoma was similar to that for lung adenocarcinoma samples with driver mutations (median of 2.94 vs. 2.51 mutations/Mb) and lower than that for lung adenocarcinoma samples without known driver mutations (median of 2.94 vs. 5.03 mutations/Mb, *P* = 0.130) from patients without IPF. Our findings suggest that not only the accumulation of somatic mutations but other factors such as inflammation and oxidative stress might contribute to the development and progression of lung cancer in patients with IPF.

## Introduction

Idiopathic pulmonary fibrosis (IPF) is characterized by chronic damage to the alveolar epithelium associated with profound changes in alveolar structure that result from abnormal tissue repair^1–3^. The disease course of IPF is variable, with progression to end-stage respiratory insufficiency and death after the onset of symptoms and diagnosis occurring in 3 to 5 years^4–6^. IPF has been implicated as an independent risk factor for the development of lung cancer, with the cumulative incidence of lung cancer ranging from 10 to 40% in IPF patients and with lung cancer being a major contributing factor to the death of such individuals^7–9^. IPF shares common risk factors with lung cancer including smoking, environmental or professional exposure, viral infection, and chronic tissue injury^1–3,10^. Although several somatic genetic alterations have been identified in lung cancer, however, the frequency of somatic mutations in IPF tissue and IPF-associated lung cancer has remained largely unknown.

Tumor mutation burden (TMB), or tumor mutation load, is defined as the number of nonsynonymous variants plus insertion and deletion variants detected per megabase (Mb) of exonic sequence. Lung cancer has a high TMB, with the large number of nonsynonymous mutations giving rise to the expression of tumor-specific neoantigens^11,12^. TMB was initially determined by whole-exome sequencing, which was not adopted in routine clinical practice because of its high cost and long turnaround time. However, TMB can now be measured by comprehensive genomic profiling or surrogate gene panel sequencing with the introduction of next-generation sequencing (NGS) assays. The Oncomine Tumor Mutation Load Assay is a targeted NGS assay that is designed for tumor profiling based on annotation of cancer driver variants and provides an accurate assessment of TMB. The assay detects and annotates low-frequency somatic variants for 409 genes distributed over 1.7 Mb of genomic DNA, with 1.2 Mb of exonic coverage, and it provides TMB values similar to those determined by whole-exome sequencing^13^. This approach may thus help to elucidate the pathogenesis of lung cancer development in individuals with lung fibrotic disease.

To explore the molecular mechanisms that underlie the development of lung cancer in the setting of IPF, we here determined TMB in pairs of fibrosing lung tissue and tumor samples from patients with IPF-associated lung adenocarcinoma and compared the obtained values with those measured for lung cancer patients without IPF. We also evaluated various factors that might influence TMB.

## Results

### Patient characteristics

We analyzed tissue specimens from 36 patients with lung adenocarcinoma. Paired samples of fibrosing lung tissue and tumor tissue were obtained from 14 patients who underwent surgical resection of lung adenocarcinoma associated with IPF. High-resolution computed tomography (HRCT) of the lung showed the usual interstitial pneumonia (UIP) pattern in each of these cases. All fibrosing lung tissue samples were also pathologically confirmed as positive for fibrosis with the UIP pattern. We also analyzed 22 lung adenocarcinoma specimens from patients without IPF, 11 of whom had a known driver mutation and 11 did not. The clinical characteristics of all the patients are shown in Table 1.

**Table 1:** Characteristics of the study patients according to idiopathic pulmonary fibrosis (IPF) and driver mutation status.

Characteristic	IPF ( +), driver mutation (–)	IPF ( −), driver mutation (–)	IPF ( −), driver mutation ( +)
	(*n* = 14)	(*n* = 11)	(*n* = 11)
Median age (range), years	74 (55–86)	70 (54–78)	64 (31–78)
**Sex**			
Male	13 (92.9)	9 (81.8)	4 (36.4)
Female	1 (7.1)	2 (18.2)	7 (63.6)
**Smoking pack-years**			
≥ 40	8 (57.1)	7 (63.6)	2 (18.2)
< 40	6 (42.9)	4 (36.4)	9 (81.8)
**Oncogenic driver**			
*EGFR* mutation	0	0	6 (54.5)
*ALK* fusion	0	0	2 (18.2)
*MET* mutation	0	0	2 (18.2)
*KRAS* mutation	0	0	1 (9.1)

Values are number (%) with the exception of age.

### TMB characterization in pairs of fibrosing lung tissue and tumor samples from patients with IPF

**Figure 1:**
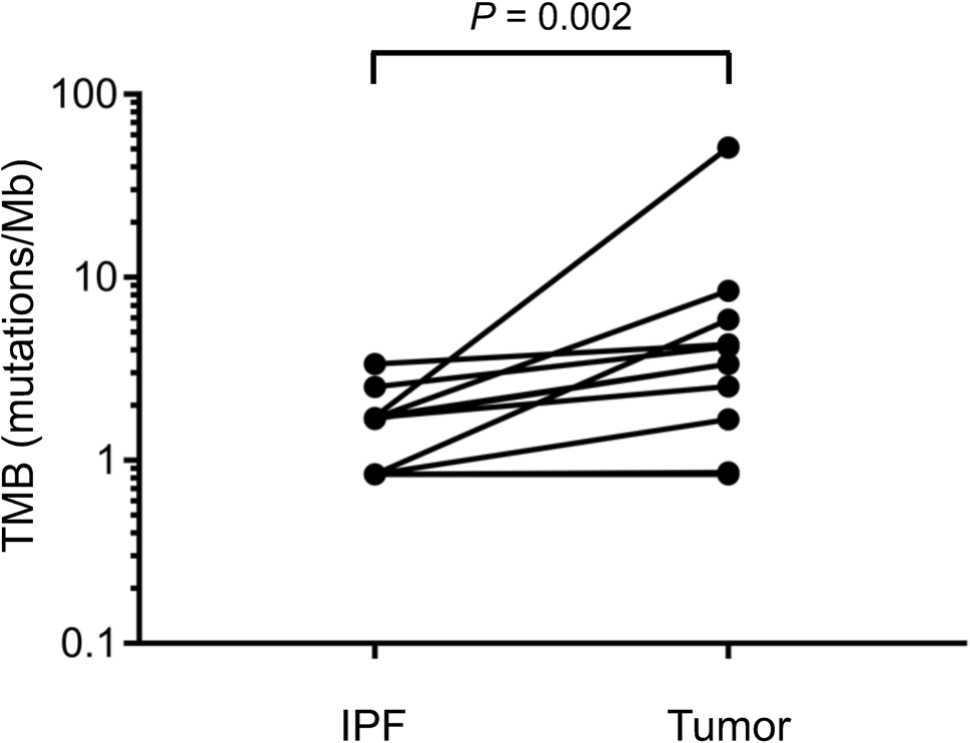
Tumor mutation burden (TMB) for matched pairs of idiopathic pulmonary fibrosis (IPF) and tumor samples from patients with IPF-associated lung adenocarcinoma. Data for three of the 14 patients with a TMB of 0 in both specimens are not shown. The *P* value was determined with the Wilcoxon matched-pairs signed-rank test.

Among the 14 patients who underwent surgical resection of lung adenocarcinoma accompanied by IPF, the median TMB in tumor tissue was significantly higher than that in fibrosing lung tissue (2.94 [range 0–51.03] versus 1.26 [range 0–3.36] mutations/Mb, *P* = 0.002) (Table 2 and Fig. 1).

**Table 2:** Tumor mutation burden (TMB) for matched pairs of fibrosing lung tissue and tumor samples.

Patient no	TMB of fibrosing lung tissue (/Mb)	TMB of tumor tissue (/Mb)
1	3.36	4.31
2	2.52	4.19
3	1.73	51.03
4	1.72	3.36
5	1.71	2.53
6	1.7	8.42
7	1.68	3.35
8	0.84	0.86
9	0.84	0.84
10	0.84	5.88
11	0.84	1.67
12	0	0
13	0	0
14	0	0
Median	1.26	2.94
Mean	1.27	6.17

### Somatic variants in pairs of fibrosing lung tissue and tumor tissue samples from patients with IPF

**Figure 2:**
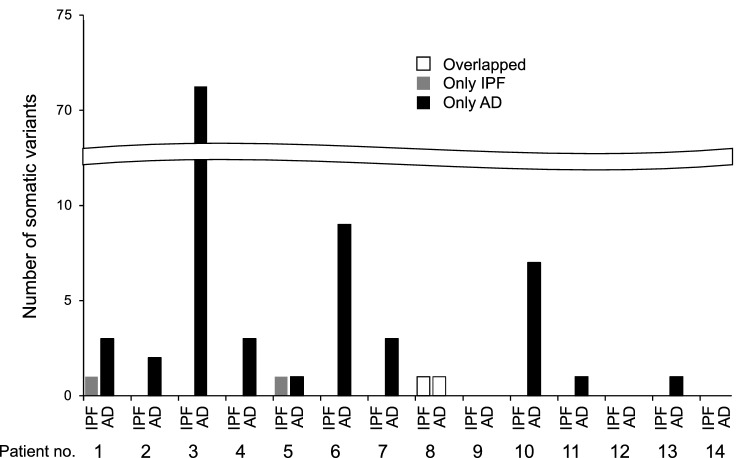
Number of somatic variants for matched pairs of idiopathic pulmonary fibrosis (IPF) and tumor samples from patients with IPF-associated lung adenocarcinoma (AD). The number of variants detected only in IPF samples, only in tumor samples, or in both sample types are indicated for each of the 14 patients.

For the 14 patients with lung cancer and IPF, NGS analysis of the fibrosing lung tissue samples detected three somatic variants in three genes (including repeat counts of those in more than one patient), with the number of somatic variants ranging from 0 to 1 for these samples (Fig. 2). In contrast, analysis of the paired tumor samples detected 102 somatic variants in 80 genes (including repeat counts of those in more than one patient), with the number of somatic variants ranging from 0 to 71 for these samples (Fig. 2). Only one variant showed overlap in one of the sample pairs (patient no. 8). No somatic variants—such as activating mutations of *EGFR*—that confer sensitivity to molecularly targeted drugs in non–small cell lung cancer were identified. Detailed information on the somatic variants detected in paired samples of fibrosing lung tissue and tumor tissue is provided in Supplementary Table S1.

### TMB characterization in tumor samples from patients with or without IPF

**Figure 3:**
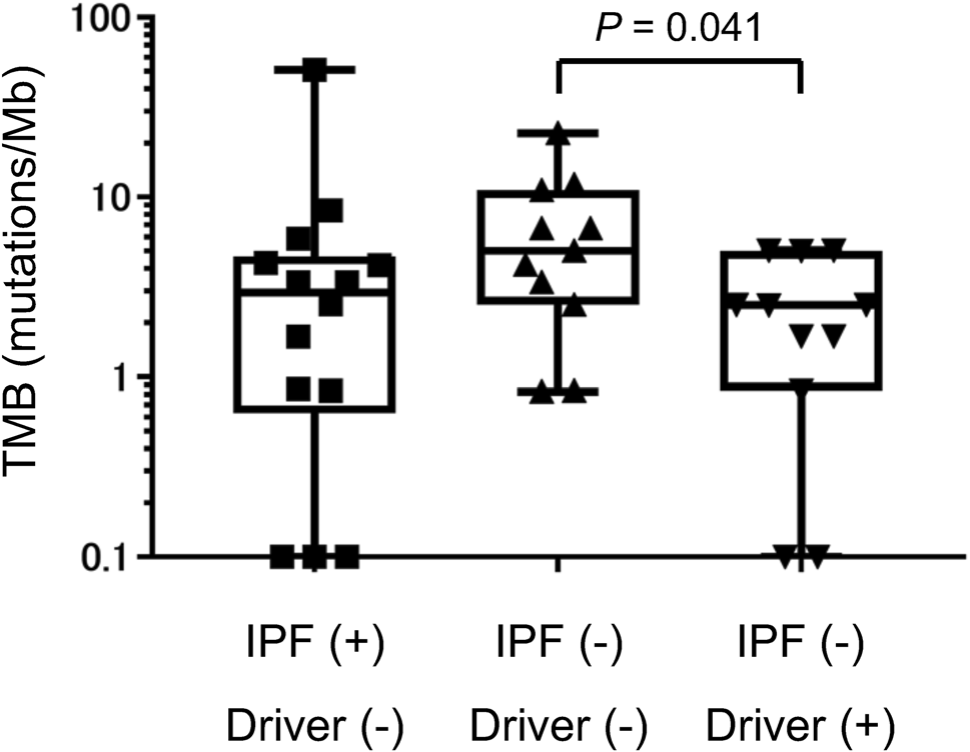
Box-and-whisker plots of tumor mutation burden (TMB) in adenocarcinoma samples from patients with idiopathic pulmonary fibrosis (IPF, *n* = 14) or without IPF and either without (*n* = 11) or with (*n* = 11) driver mutations. The boxes indicate the median and upper and lower quartiles, and the whiskers represent the range. The *P* value was determined with the Mann–Whitney U test.

We also analyzed TMB in lung adenocarcinoma samples from 22 patients without IPF. The median TMB for the 11 of these patients without known driver mutations was significantly higher than that for the 11 individuals with such mutations (5.03 [range 0.83–22.67] vs. 2.51 [range 0–5.05] mutations/Mb, *P* = 0.041) (Fig. 3). The median TMB in the tumor samples without driver mutations from non-IPF patients was also higher than that in those from IPF patients, although this difference did not achieve statistical significance (*P* = 0.130) (Fig. 3).

### Relation between smoking pack-years and TMB in tumor samples from patients with or without IPF

**Figure 4:**
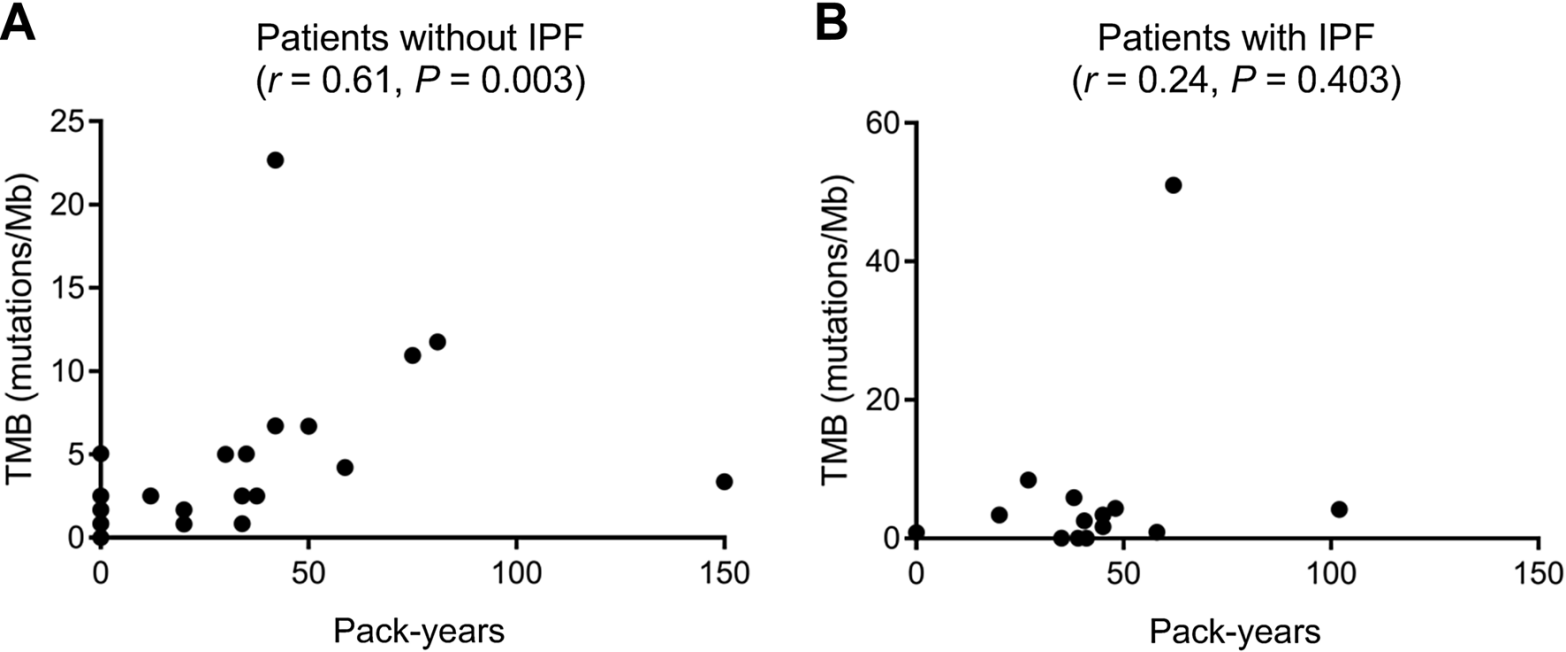
Correlation analysis for smoking pack-years and tumor mutation burden (TMB) in tumor samples. (**A**) Correlation between pack-years and TMB in tumor samples from patients without idiopathic pulmonary fibrosis (IPF, *n* = 22). (**B**) Correlation between pack-years and TMB in tumor samples from patients with IPF (*n* = 14). Spearman’s correlation coefficient (*r*) and *P* values are indicated.

Smoking, age, and male sex are common risk factors for IPF and lung cancer. We assessed the possible influence of these factors on TMB in tumor samples from patients with or without IPF. A positive correlation was detected between smoking pack-years and TMB in tumor samples from patients without IPF (*r* = 0.61, *P* = 0.003) (Fig. 4A), whereas such a correlation was not apparent for patients with IPF (*r* = 0.24, *P* = 0.403) (Fig. 4B). Correlations between age or sex and TMB in tumor specimens from patients with or without IPF were also not observed (data not shown).

## Discussion

Previous characterization of TMB in patients with lung cancer accompanied by IPF has been limited. Although TMB was evaluated in IPF-associated lung cancer by whole-exome sequencing^14^, it has not been examined in IPF tissue from patients with IPF-associated tumors. With the use of an NGS assay, we have here investigated TMB and somatic variants in both fibrosing lung tissue and tumor samples from patients with IPF-associated lung adenocarcinoma. We found that TMB of the tumor samples was significantly higher than that of the paired fibrosing lung tissue samples, suggesting that the accumulation of somatic mutations contributes to tumorigenesis in IPF tissue.

Genetic alterations such as those in *TP53*, *HER2*, and *KRAS* associated with non–small cell lung cancer were found to overlap with those in squamous metaplasia or atypical alveolar hyperplasia, indicating that such metaplasia and hyperplasia are preneoplastic lesions^15–17^. On the other hand, with the use of a panel covering a relatively small number of genes (161 genes), we previously found that somatic alterations were not frequently shared between lung tumor and corresponding IPF tissue^18^. Although we adopted a panel covering a greater number of genes (409 genes) in the present study, few somatic variants were detected in the IPF samples, and, with the exception of one variant, these did not overlap with variants detected in paired tumor samples.

Lung cancer accompanied by IPF has been thought to have a high TMB because IPF is associated with smoking history, male sex, and microsatellite instability, all of which are related to a high mutation burden^19,20^. We have now found that TMB in adenocarcinoma samples from patients with IPF was similar to that in those with driver mutations from patients without IPF and lower than that in those without driver mutations from non-IPF patients, consistent with previous observations^14^. Our results suggest that various factors such as epigenetic alterations, abnormal expression of microRNAs, the activation of signaling pathways, and other cellular or molecular changes as well as genetic mutations might be responsible for development and progression of lung cancer in IPF patients^2,10^.

Tobacco smoking is also a major risk factor for lung cancer^21^, which confounds exploration of the relation between IPF and lung cancer. However, a population-based cohort study in the United Kingdom suggested that the incidence of lung cancer is increased independently of smoking history in IPF patients^22^. Moreover, various factors other than smoking, older age, male sex, and coexisting emphysema are also strong risk factors for the development of lung cancer in individuals with IPF^10^. In the present study, we detected a positive correlation between smoking pack-years and TMB in tumor samples for patients without IPF, but not for those with IPF. These results indicate that, although genetic alterations induced by smoking may be related to the development of lung cancer in non-IPF patients, other factors such as chronic inflammation may play a more important role in this process in individuals with IPF.

There are several limitations to the present study. First, the study population was relatively small. Patients at an inoperable stage or with poor pulmonary function could not be included in the study because they could not be actively diagnosed or treated, as a result of an increased risk of IPF-associated life-threatening complications of the diagnostic procedure or treatment. Second, the obtained genomic profiles were derived from limited target sequencing without gene expression or epigenetic data. Analysis of other genes, such as those related to the pulmonary surfactant system or cell death signaling might also provide insight into the etiology of IPF-associated lung cancer^23,24^. In addition, the Oncomine Tumor Mutation Load Assay adopted in the present study has not been validated in lung tissue, with the result that the data should be interpreted with caution.

TMB is an emerging biomarker for the response to immune checkpoint inhibitors^25,26^. Evidence suggests that TMB is a surrogate for tumor neoantigens recognizable by the adaptive immune system, which targets and eliminates tumor cells on detection of such neoantigens^27^. We have now found that TMB in adenocarcinoma associated with IPF was lower than that in driver mutation–negative adenocarcinoma not accompanied by IPF.

In summary, as far as we are aware, our study is the first to investigate TMB in pairs of fibrosing lung tissue and tumor samples from patients with IPF-associated lung adenocarcinoma. We found that the TMB of IPF-associated lung cancer was low and was not correlated with smoking status. We also found that somatic alterations were rarely shared between tumor and corresponding IPF tissue. Our findings thus suggest that not only the accumulation of somatic mutations but also various other factors such as inflammation and oxidative stress might be responsible for the development and progression of lung cancer in patients with IPF. Further comprehensive analysis including at the epigenetic and cell and molecular biology levels is warranted to obtain further insight into the etiology of IPF-associated lung cancer.

## Methods

### Patients

Specimens from 36 patients with lung adenocarcinoma were analyzed. Fourteen patients who underwent surgical resection for lung adenocarcinoma associated with pathologically diagnosed IPF at Kyushu University Hospital or Kyushu Cancer Center between January 2012 and December 2018 and for whom tumor tissue specimens were available were thus enrolled in the study, as were 22 patients diagnosed with lung adenocarcinoma but without IPF at Kyushu University Hospital between January 2014 and December 2019. The clinical characteristics, pathological data, tumor genotype, and HRCT imaging pattern of IPF were extracted for the study patients by retrospective chart inspection. IPF was defined on the basis of the HRCT and pathological criteria outlined in the joint statement of the American Thoracic Society, European Respiratory Society, Japanese Respiratory Society, and Latin American Thoracic Association^3^. The study was approved by the Ethics Committees of Kyushu University Hospital and Kyushu Cancer Center and was conducted in accordance with the Declaration of Helsinki. This research was defined as a study with human samples according to the guidelines of the Ministry of Health, Labor, and Welfare of Japan. All methods were performed in accordance with the relevant guidelines. Informed consent was obtained from all subjects.

### Tumor mutation load assay

The Oncomine Tumor Mutation Load Assay is a polymerase chain reaction (PCR)–based NGS assay designed for tumor profiling by annotation of cancer driver variants and provides an assessment of TMB. The assay detects and annotates low-frequency somatic variants, including missense and nonsense single nucleotide variants (SNVs) and insertion and deletion variants (INDELs), for 409 genes spanning ~ 1.7 Mb of genomic DNA, encompassing 1.2 Mb of exonic sequence^14^. DNA was isolated from freshly frozen specimens of fibrosing lung tissue and lung tumor tissue, and the isolated material (10 ng) was subjected to multiplex PCR-based amplification with the use of an Ion AmpliSeq Library Kit Plus (Thermo Fisher Scientific) according to the standard protocol. Sequencing was performed with a high-throughput semiconductor sequencing platform, the Ion GeneStudio S5 System, and with a 540 chip in order to achieve high median coverage (> 500 ×) and uniformity (> 90%). Reads were aligned to the hg19 human genome with the use of Torrent Suite 5.6, and BAM files are transferred to Ion Reporter 5.6 for variant calling and secondary analysis including TMB calculation. For comparison of somatic variants between paired samples of fibrosing lung tissue and tumor tissue, somatic variants with an allele frequency of ≥ 30% in both fibrosing lung tissue and tumor samples were excluded because of the possibility of their being germ-line variants.

### **Statistical analysis**

The difference in TMB between fibrosing lung tissue and paired tumor samples was evaluated with the Wilcoxon matched-pairs signed-rank test. The difference between TMB in tumor samples from patients with IPF and that in those from patients without IPF was assessed with the Mann–Whitney U test. The relation between TMB and smoking pack-years was examined with Spearman’s rank correlation. All *P* values shown are two-sided, and those of < 0.05 were considered statistically significant. All statistical analysis was performed with GraphPad Prism 9.

## Supplementary Information

Supplementary Information.

## Data Availability

All data generated or analysed in this study are included in this published article.
