# Supplementary material for: Paired analysis of tumor mutation burden for lung adenocarcinoma and associated idiopathic pulmonary fibrosis
Source: Sci Rep. 2021 Jun 17;11:12732. doi: 10.1038/s41598-021-92098-y (PMC8211684; doi:10.1038/s41598-021-92098-y)
Supplement: Supplementary file 1 — Supplementary Information. [file 41598_2021_92098_MOESM1_ESM.docx]

**Table S1.** Detailed information for somatic alterations detected in matched idiopathic pulmonary fibrosis (IPF, *n* = 14) or tumor (*n* = 14) samples by NGS

| Patient | Tissue | Gene | Location | Type | Coding site | Variant allele frequency (%) |  |
| --- | --- | --- | --- | --- | --- | --- | --- |
| 1 | IPF | *HLF* | UTR | INDEL | c.-47T>TCTTTT | 5.45 |  |
|  | Tumor | *ARNT* | Exonic | SNV | c.1241A>G | 6.69 |  |
|  |  | *MET* | Exonic | SNV | c.2080G>T | 8.76 |  |
|  |  | *PTPRT* | Intronic | SNV | c.2548+28G>A | 8.01 |  |
| 2 | IPF | Not detected | | | | | |
|  | Tumor | *CDH2* | Exonic | SNV | c.1681C>T | 9.29 |  |
|  |  | *ITGB2* | Exonic | SNV | c.2276C>T | 12.92 |  |
| 3 | IPF | Not detected | | | | | |
|  | Tumor | *ACVR2A* | Intronic | SNV | c.56-4A>T | 27.99 |  |
|  |  | *ADAMTS20* | Intronic | SNV | c.5447-17T>C | 23.61 |  |
|  |  | *ADAMTS20* | Exonic | SNV | c.2023G>C | 5.34 |  |
|  |  | *ADGRL3* | Intronic | SNV | c.379+42C>A | 11.66 |  |
|  |  | *ADGRL3* | Exonic | SNV | c.450G>A | 23.38 |  |
|  |  | *ALK* | Intronic | SNV | c.2355+62G>T | 8.9 |  |
|  |  | *ARID1A* | Exonic | SNV | c.1435C>T | 19.19 |  |
|  |  | *ARID2* | Exonic | SNV | c.3580C>G | 6.36 |  |
|  |  | *ATR* | Exonic | SNV | c.5399G>T | 22.73 |  |
|  |  | *BMPR1A* | Exonic | SNV | c.960C>A | 29.23 |  |
|  |  | *BRIP1* | Intronic | SNV | c.2905+62G>C | 19.78 |  |
|  |  | *CDK6* | Exonic | SNV | c.195G>C | 5.68 |  |
|  |  | *CDK8* | Exonic | SNV | c.303A>G | 25.36 |  |
|  |  | *CSMD3* | Intronic | SNV | c.9862+53G>T | 19.54 |  |
|  |  | *CSMD3* | Intronic | INDEL | c.8614+22CA>A | 19.11 |  |
|  |  | *DPYD* | Exonic | SNV | c.2455A>G\| | 14.42 |  |
|  |  | *DST* | Exonic | SNV | c.10096G>C | 15.97 |  |
|  |  | *EGFR* | Exonic | SNV | c.695G>T | 19.92 |  |
|  |  | *EP300* | Exonic | SNV | c.56C>T | 6.9 |  |
|  |  | *EP400* | Splice site | SNV | c.1930-2A>G | 16.75 |  |
|  |  | *EP400* | Intronic | SNV | c.3924-31G>A | 19.38 |  |
|  |  | *EPHB1* | Intronic | SNV | c.2690+5G>A | 7.58 |  |
|  |  | *ERBB4* | Intronic | SNV | c.1871+10C>T | 14.21 |  |
|  |  | *ERBB4* | Intronic | SNV | c.1490-84C>G | 6.68 |  |
|  |  | *ERCC4* | Exonic | SNV | c.613G>T | 15.14 |  |
|  |  | *ERG* | Exonic | SNV | c.430G>C | 15.98 |  |
|  |  | *EZH2* | Exonic | SNV | c.697G>C | 19.05 |  |
|  |  | *FANCA* | Exonic | SNV | c.1483C>T | 17.29 |  |
|  |  | *FLI1* | Exonic | SNV | c.1269G>T | 20.92 |  |
|  |  | *FLT3* | Exonic | SNV | c.2285C>G | 7.52 |  |
|  |  | *GRM8* | Exonic | INDEL | c.2143delG | 29.7 |  |
|  |  | *HOOK3* | Exonic | SNV | c.265G>C | 21.19 |  |
|  |  | *IKZF1* | Exonic | SNV | c.959A>T | 23.84 |  |
|  |  | *IL6ST* | Exonic | SNV | c.36G>T | 28.7 |  |
|  |  | *JAK1* | Exonic | SNV | c.29G>T | 17.79 |  |
|  |  | *KAT6B* | Exonic | SNV | c.5294A>G | 8 |  |
|  |  | *KEAP1* | Exonic | SNV | c.851A>T | 26.94 |  |
|  |  | *KMT2C* | Exonic | SNV | c.6382C>G | 5.79 |  |
|  |  | *KMT2D* | Exonic | SNV | c.9043G>T | 16.86 |  |
|  |  | *KMT2D* | Intronic | SNV | c.8047-16T>C | 9.25 |  |
|  |  | *KNL1* | Exonic | SNV | c.824G>C | 20.2 |  |
|  |  | *LRP1B* | Intronic | SNV | c.8149+52G>T | 28.48 |  |
|  |  | *LRP1B* | Exonic | SNV | c.6412C>T | 13.27 |  |
|  |  | *LRP1B* | Exonic | SNV | c.1594C>A | 15.93 |  |
|  |  | *MAP2K4* | Exonic | SNV | c.870C>T | 9.07 |  |
|  |  | *MDM4* | Exonic | SNV | c.1004C>G | 18.49 |  |
|  |  | *MRE11* | Exonic | SNV | c.1823C>T | 17.58 |  |
|  |  | *MTRR* | Exonic | SNV | c.56G>T | 28.68 |  |
|  |  | *MYH9* | Intronic | SNV | c.869-71A>G | 7.18 |  |
|  |  | *NF1* | Exonic | SNV | c.170G>T | 20.91 |  |
|  |  | *NIN* | Exonic | SNV | c.3213G>C | 5.39 |  |
|  |  | *PDGFRA* | Exonic | SNV | c.2022A>T | 25.84 |  |
|  |  | *PDGFRA* | Exonic | SNV | c.3002A>T | 18.93 |  |
|  |  | *PDGFRB* | Exonic | SNV | c.1866G>A | 13.41 |  |
|  |  | *PGAP3* | Intronic | SNV | c.433-12C>T | 20 |  |
|  |  | *PIK3C2B* | Intronic | SNV | c.4398+73C>G | 5.16 |  |
|  |  | *PIK3CA* | Intronic | SNV | c.1251+12C>G | 11.8 |  |
|  |  | *PLEKHG5* | Exonic | SNV | c.1890C>T | 14.84 |  |
|  |  | *PRKDC* | Intronic | SNV | c.11578+73C>G | 25.79 |  |
|  |  | *PRKDC* | Intronic | SNV | c.11578+43C>G | 25.27 |  |
|  |  | *PTPRT* | Exonic | SNV | c.2862T>A | 12.92 |  |
|  |  | *RET* | Exonic | SNV | c.1185G>T | 16.13 |  |
|  |  | *RNF213* | Exonic | SNV | c.1253G>C | 8.45 |  |
|  |  | *RNF213* | Exonic | SNV | c.8751C>T | 9.23 |  |
|  |  | *ROS1* | Exonic | SNV | c.6416C>T | 29.47 |  |
|  |  | *STK36* | Exonic | SNV | c.2269G>C | 14.4 |  |
|  |  | *TBX22* | Intronic | SNV | c.175+34G>A | 22.05 |  |
|  |  | *TNK2* | Exonic | SNV | c.1503C>T | 12.51 |  |
|  |  | *TRIP11* | Exonic | SNV | c.4715A>T | 21.33 |  |
|  |  | *UBR5* | Intronic | SNV | c.7601+17C>G | 15.52 |  |
|  |  | *ZNF521* | Exonic | SNV | c.1265A>C | 6.58 |  |
| 4 | IPF | Not detected | | | | | |
|  | Tumor | *MUC1* | Exonic | SNV | c.640A>G | 6.71 |  |
|  |  | *EXT1* | Intronic | SNV | c.1883+52G>T | 6.89 |  |
|  |  | *PIK3CA* | Exonic | SNV | c.3127A>T | 7.92 |  |
| 5 | IPF | *HLF* | UTR | INDEL | c.-47T>TCTTTT | 7.5 |  |
|  | Tumor | *CARD11* | Exonic | SNV | c.489G>C | 5.03 |  |
| 6 | IPF | Not detected | | | | | |
|  | Tumor | *BCL6* | Intronic | INDEL | c.1977+66G>GGGCAGGGAAGGAGGTGGAG | 9.84 |  |
|  |  | *CBL* | Exonic | SNV | c.342T>G | 15.21 |  |
|  |  | *CDH2* | Exonic | SNV | c.1547T>A | 12.97 |  |
|  |  | *DST* | Exonic | SNV | c.11151G>C | 12.81 |  |
|  |  | *EP300* | Exonic | SNV | c.1678G>C | 9.95 |  |
|  |  | *KMT2A* | Exonic | SNV | c.7373G>C | 29.15 |  |
|  |  | *PIK3CA* | Exonic | SNV | c.746A>C | 26.73 |  |
|  |  | *THBS1* | Intronic | SNV | c.3506-64G>C | 13.45 |  |
|  |  | *UBR5* | Exonic | SNV | c.6919G>A | 28.73 |  |
| 7 | IPF | Not detected | | | | | |
|  | Tumor | *LRP1B* | UTR | INDEL | c.-70C>GCC | 29.6 |  |
|  |  | *SMO* | Exonic | SNV | c.1558A>G | 23.65 |  |
|  |  | *TP53* | Exonic | SNV | c.388C>G | 16.53 |  |
| 8 | IPF | *PDE4DIP* | Exonic | SNV | c.3978C>G | 21.95 |  |
|  | Tumor | *PDE4DIP* | Exonic | SNV | c.3978C>G | 19.92 |  |
| 9 | IPF | Not detected | | | | | |
|  | Tumor | Not detected | | | | | |
| 10 | IPF | Not detected | | | | | |
|  | Tumor | *ARID1A* | Exonic | SNV | c.2132C>G | 13.15 |  |
|  |  | *BIRC5* | Exonic | SNV | c.325T>G | 7.17 |  |
|  |  | *CDKN2A* | Exonic | INDEL | c.284_313delTGGTGCTGCACCGGGCCGGGGCGCGGCTGG | 6.16 |  |
|  |  | *PKHD1* | Exonic | SNV | c.5573T>C | 7.2 |  |
|  |  | *PTEN* | Exonic | SNV | c.380G>T | 8.25 |  |
|  |  | *PTPRT* | Intronic | SNV | c.1762+61C>G | 9.98 |  |
|  |  | *SYNE1* | Exonic | SNV | c.8879G>T\| | 9.04 |  |
| 11 | IPF | Not detected | | | | | |
|  | Tumor | *PIK3CA* | Exonic | SNV | c.1624G>A | 6.37 |  |
| 12 | IPF | Not detected | | | | | |
|  | Tumor | Not detected | | | | | |
| 13 | IPF | Not detected | | | | | |
|  | Tumor | *ARID2* | Splice site | SNV | c.1716-2A>T | 13.33 |  |
| 14 | IPF | Not detected | | | | | |
|  | Tumor | Not detected | | | | | |

UTR, untranslated region; INDEL, insertion or deletion; SNV, single nucleotide variant.
